# DNA Methyltransferase Inhibitors Improve the Effect of Chemotherapeutic Agents in SW48 and HT-29 Colorectal Cancer Cells

**DOI:** 10.1371/journal.pone.0092305

**Published:** 2014-03-27

**Authors:** Sylwia Flis, Agnieszka Gnyszka, Krzysztof Flis

**Affiliations:** 1 Department of Pharmacology, National Medicines Institute, Warsaw, Poland; 2 Department of Genetics, Institute of Biochemistry and Biophysics, Polish Academy of Sciences, Warsaw, Poland; Institute of Clinical Physiology, c/o Toscana Life Sciences Foundation, Italy

## Abstract

DNA methylation is an epigenetic phenomenon known to play an important role in the development and progression of human cancer. Enzyme responsible for this process is DNA methyltransferase 1 (DNMT1) that maintains an altered methylation pattern by copying it from parent to daughter DNA strands after replication. Aberrant methylation of the promoter regions of genes critical for normal cellular functions is potentially reversible. Therefore, inactivation of DNMT1 seems to be a valuable target for the development of cancer therapies. Currently, the most popular DNMT inhibitors (DNMTi) are cytidine analogues like 5-azacytidine, 5-aza-2′-deoxycytidine (decitabine) and pyrimidin-2-one ribonucleoside (zebularine). In colorectal cancer, epigenetic modifications play an essential role at each step of carcinogenesis. Therefore, we have addressed the hypothesis that DNA methyltransferase inhibitors may potentiate inhibitory effects of classical chemotherapeutic agents, such as oxaliplatin and 5-fluorouracil (5-FU), commonly used in colorectal cancer therapy. Here, our report shows that DNMTi can have positive interactions with standard chemotherapeutics in colorectal cancer treatment. Using pharmacological models for the drug-drug interaction analysis, we have revealed that the combination of decitabine with 5-FU or oxaliplatin shows the most attractive interaction (synergism), whereas the effect of zebularine in combinations with chemotherapeutics is moderate and may be depended on genetic/epigenetic background of a cell line or secondary drug used in combination. Our results suggest that DNMTi administered in combination with standard chemotherapeutics might improve the treatment of patients with colorectal cancers.

## Introduction

Colorectal cancer (CRC) is the second most common cancer in the non-smoking population worldwide. It is estimated that over 600000 people die from it globally each year [1]. It means that colorectal cancer is a leading cause of cancer related deaths. Unfortunately, CRC develops for a long time without any symptoms; therefore the disease is recognized at advanced stages. Generally, the risk of CRC increases with age and is caused not only by genetic alterations involving oncogenes and tumor suppressor genes, but is also driven by epigenetic alterations involving changes in gene expression patterns, which are not dependent on changes in the DNA sequence. One of the epigenetic events is caused by DNA methyltransferases (DNMTs), which catalyze the covalent addition of the methyl group to the 5′ position of cytosine in the CpG dinucleotide from the donor *S*-adenosylmethionine. Cytosine methylation occurs in genomic regions called CpG islands and it is known to alter the chromatin structure leading to gene silencing. During colorectal cancer progression, a global genome demethylation coupled with selective hypermethylation of tumor suppressors, cell cycle regulators and proapoptotic genes is observed [2]. Because epigenetic modifications are potentially reversible, they constitute an interesting therapeutic strategy with use of DNMT inhibitors (DNMTi).

Decitabine and zebularine are DNMT inhibitors, which may potentially reverse epigenetic alterations resulting in reactivation of silenced genes, blocking cancer cell proliferation and/or inducing apoptosis [3], [4]. Both agents seem to be very interesting and valuable for therapy of solid tumors. Decitabine, a cytidine analog, has been approved by the U.S. Food and Drug Administration (FDA) for the treatment of hematological malignancies [5], whereas zebularine in comparison to other cytidine analogs is more stable, less toxic and can be orally administered [6].

It appears to be reasonable to use demethylating agents in combination with chemotherapeutic agents. Interestingly, encouraging results were obtained with combination of decitabine and carboplatin in patients with solid tumors [7]. The authors concluded that decitabine combines safely with carboplatin and that the regimen causes epigenetic changes. In another phase I study, a combination of cisplatin with decitabine resulted in one partial response in patient with cervical cancer and two minor responses: one in patient with non-small-cell lung cancer and the other in patient with cervical cancer [8]. However, despite lines of evidence indicating that demethylating agents might improve anticancer activity of classic chemotherapeutic agents, the knowledge for using them for solid tumors is still insufficient and needs to be further evaluated.

Therefore, to test this hypothesis, the CRC cell line survival model was chosen to study these interactions. With the help of this model and pharmacological analysis we have designed the study to find out the outcome of the interaction between DNMT inhibitors, decitabine or zebularine, and classic anticancer chemotherapeutics used in the treatment of CRC such as oxaliplatin, an inter- and intra-DNA cross-linking agent, and 5-fluorouracil, a thymidylate synthase inhibitor. The studies of interactions between chemotherapeutic agents and DNMTi agents seem to be reasonable, because all these compounds have been tested for their cytotoxic properties against normal cells [5], [9], [10] and all of them except zebularine are approved by the U.S. FDA for medical use.

## Materials and Methods

### Cell culture and drug treatment

As a model of colon cancer cells, the HT-29 and SW48 human colorectal cancer cell lines, obtained from American Type Culture Collection (ATCC, Manassas, VA, USA), were used. The cells were cultured in RPMI 1640 medium (Gibco, Paisley, UK) supplemented with 10% (v/v) heat-inactivated fetal bovine serum (FBS, Gibco), 2 mM glutamax (Gibco), 100 units/ml penicillin, 100 μg/ml streptomycin and 250 ng/ml amphoterycin (Gibco) at 37°C in a humidified atmosphere including 5% CO_2_. Cells were incubated with the drugs for 24, 48 and 72 h, but only results observed following the 72 h treatment are presented as their positive become more pronounced.

### Drugs

The following drugs were studied: 5-fluorouracil (5-FU), oxaliplatin, zebularine and decitabine (Sigma, St. Louis, MI, USA). The concentrations of the drugs were in the range up to 100 μM. All agents were dissolved in dimethyl sulfoxide Hybri-Max (DMSO, Sigma) and then diluted in the media for experiments. The final concentration of DMSO, without affecting cell survival, was maintained at 0.2%. In all experiments, control cells were incubated with DMSO.

### MTT assay

The assay relies on the ability of viable cells to metabolically reduce a yellow tetrazolium salt to a purple formazan product. This reaction requires active mitochondrial reductase enzymes.

Cells were grown in 96-well plates (1×10^4^ cells/200 μl/well). After incubation with the reagents, the medium was removed and the cells were treated with 50 μl of 3-(4,5-dimethylthiazol-2-yl)-2,5-diphenyltetrazolium bromide solution (MTT, Sigma) for 4 h at 37°C. Next, 150 μl of solubilization solution (10% sodium dodecyl sulfate, SDS) was added and the mixture was incubated at 37°C overnight. The solubilized formazan product was spectrophotometrically quantified using a microtiter plate reader, Power Wave XS (Bio-Tek, Winooski, VT, USA), at 570 nm wavelength.

### Analysis of drug interactions

Cells of SW48 and HT-29 cell lines were simultaneously incubated for 72 h with chemotherapeutic agents and DNMT inhibitors or with each agent alone. The nature of the interactions between drugs studied was analyzed with the help of izobologram [11] and combination-index (CI) methods, derived from the median-effect principle of Chou and Talalay [12], [13]. The data obtained from the cytotoxicity experiments were used for mathematical and quantitative evaluation of drug-drug interactions.

Isoboles were defined by effects of the paired drugs studied. The effects obtained by the half maximal inhibitory concentration (IC_50_) of either drug within the pair formed the basis for the additivity line; synergism or antagonism was present, when the same effect was obtained by the combination of drugs in lower or higher doses, respectively.

A commercial software package, CalcuSyn ver. 2.0 (Biosoft, Cambridge, United Kingdom), was used for median-effect analysis. We calculated the CI values based on the formula: CI  =  (D)_1_/(D_x_)_1_ + (D)_2_/(D_x_)_2_ for mutually exclusive drugs. In the denominator, (D_x_) is for D_1_ “alone” that inhibits a system *x*%, and (D_x_)_2_ is for D_2_ “alone” that inhibits a system *x*%. In the numerators, (D)_1_ + (D)_2_ “in combination” also inhibit *x*%. CI values were generated at different effect levels (Fa, the fraction affected by D, i.e. percentage inhibition/100) from 0.05 to 0.95 (5–95% cell kill). Synergy is indicated by CI values <1, additivity by CI values  =  1 and antagonism by CI values >1.

### Flow cytometry analysis

For cell cycle analysis the cells (∼1×10^6^) were suspended in 4 ml of 80% ethanol (−20°C) and incubated at −20°C for 24 h, washed twice in phosphate-buffered saline (PBS), and stained with 50 μg/ml propidium iodide (PI) and 100 μg/ml RNase in 0.1% PBST solution (PBS supplemented with 0.1% Triton X–100) for 30 min in the dark at 4°C. The samples were then measured using a BD FACSCalibure flow cytometer (BD Biosciences, San Jose, CA, USA). The DNA histograms were analyzed using ModFit software (BD Biosciences).

Apoptosis of the cells was measured, according to the manufacturer's instructions, using an annexin V-FITC kit (BD Biosciences). The cells were collected after treatment, washed twice with PBS and centrifuged. The cell pellet was resuspended in ice-cold binding buffer. The annexin V-FITC and PI solutions were added to the cell suspension and gently mixed. The samples were then incubated for 15 min in the dark before flow cytometry analysis.

For immunofluorescent staining, cells were fixed in 1.5% formaldehyde for 10 min at room temperature (RT) and permeabilized with cold methanol for 20 min at 4°C. After washing, cells were incubated with desire primary antibody (Cell Signaling Technology, Danvers, MA, USA) against phospho-ATR [(P)-Ser428, Cat. No. 2853] and phospho-ATM [(P)-Ser1981, Cat. No. 5883] at 1:100 dilution in 0.5% BSA/PBS for 1 h at RT. After washing with PBS, appropriate secondary antibody conjugated with FITC (Santa Cruz Biotechnology, Dallas, TX, USA) were added at 1:500 dilution in 0.5% BSA/PBS and incubated for 30 min at RT. After washing, the cells were analyzed by flow cytometry.

For all applications 10,000 events per sample were analyzed by fluorescence-activated cell sorting (FACS).

### Semi-quantitative RT-PCR

The mRNA levels of *CCNE1*, *ATM* and *GAPDH* were analyzed by RT-PCR using total RNA from HT-29 and SW48 cells isolated using the GenElute™ Mammalian Total RNA Miniprep Kit (Sigma), as described by the manufacturer. One hundred ng of total RNA was used in the reverse transcription reaction with Omniscript Reverse Transcriptase (Qiagen, Hilden, Germany) and oligo (dT)_18_ primer (Fermentas, Vilnius, Lithuania). The PCR amplifications were performed in a 50 μl total volume according to manufacturer's instruction using HotStarTaq Master Mix (Qiagen), 3 μl of cDNA as a template and the following primers pairs: *CCNE1* (5'-AACTCAACGTGCAAGCCTCG-3', 5'-CATCTCCTGAACAAGCTCCA-3'), *ATM* (5'-GCCTTGAGTCTGTGTATTCG-3', 5'-CCACTCAGAGACTCCACAGC-3') and *GAPDH* (5'-TCACCATCTTCCAGGAGCGA-3', 5'-TGGTCATGAGTCCTTCCACG-3'). The *GAPDH* mRNA levels were used as internal controls. The amplified fragments were separated on 2% agarose gels, stained with ethidium bromide and photographed under UV light.

### Preparation of protein lysates and Western blotting

The cells were washed with cold PBS buffer and then proteins from five cellular compartments were isolated using the Subcellular Protein and Fractionation Kit for Cultured Cells (Pierce, Rockford, IL, USA). Protein concentration in the samples was measured using BCA protein assay kit (Pierce). Samples containing 60 μg of protein were denatured and fractionated by 7, 12 or 15% SDS-PAGE. After electrophoresis, the proteins were transferred onto a nitrocellulose membrane and probed with anti-human antibodies specific to: cyclin A1 and D1, PARP, caspase-3 and -8 (Santa Cruz Biotechnology); p21 (Cat. No. 554228), p53 (Cat. No. 610183), Bax (Cat. No. 610982, BD Biosciences); β-actin (Cat. No. A1978, Sigma); and the DNA Damage Antibody Sampler Kit (phospho-Chk1 [(P)-Ser296], phospho-Chk2 [(P)-Thr68], phospho-histone H2A.X [γH2A.X, (P)-Ser139], phospho-p53 [(P)-Ser15], and phospho-BRCA1 [(P)-Ser1524]; Cat. No. 9947; Cell Signaling Technology). All antibodies in the DNA Damage Antibody Sampler Kit recognize their targets proteins only when modified at the indicated sites. Therefore, antibodies against unmodified proteins were not used.

Anti-Bax antibody recognizes human Bax-α form. An alternative splicing of Bax pre-mRNA produces the integral membrane form Bax-α and the two cytosolic forms β and γ. This antibody is recommended by BD company for detection of apoptosis. Anti-p53 antibody recognizes the C terminal region of the protein (the 195–393 a.a. was used as an antigen) and is able to recognize both the wild-type and R273H forms of p53. This antibody is also recommended by BD company for detection of apoptosis.

The signal on blots was detected by a colorimetric method using the CN/DAB Substrate Kit (Pierce) and SignalBoost Immunoreaction Enhancer Kit (Calbiochem, San Diego, CA, USA).

### Mitochondrial membrane potential (ΔΨ_m_) measurement

The Δ*Ψ_m_* was measured by flow cytometry using 10 mg/ml of 5,5′,6,6′-tetrachloro-1,1′,3,3′-tetraethylbenzimidazolo- carbocyanine iodide (JC-1 dye, Sigma), which stains mitochondria in living cells. In healthy cells, the dye accumulates in mitochondria, forming aggregates that emit red fluorescence, while in apoptotic cells the dye remains in monomeric form in cytoplasm and emits green fluorescence. Cells were treated with chemotherapeutics, DNMT inhibitors or their combinations for 72 h and stained as described by Mahyar-Roemer *et al.* [14] and then examined by FACSCalibure flow cytometer. Mitochondria containing red JC-1 aggregates in healthy cells were detectable in FL2 channel, while green JC-1 monomers in apoptotic cells were detectable in FL1 channel.

### Statistical analysis

Data are presented as mean values ±SD. Statistical comparisons among groups were performed by Student's t-test or one-way analysis of variance (ANOVA) followed by Tukey *post hoc* test. Significance was assumed at *P* < 0.05 (marked with asterisks on graphs).

## Results

### Growth studies and effects of combination treatments of chemotherapeutic agents with DNMTi

**Figure 1:**
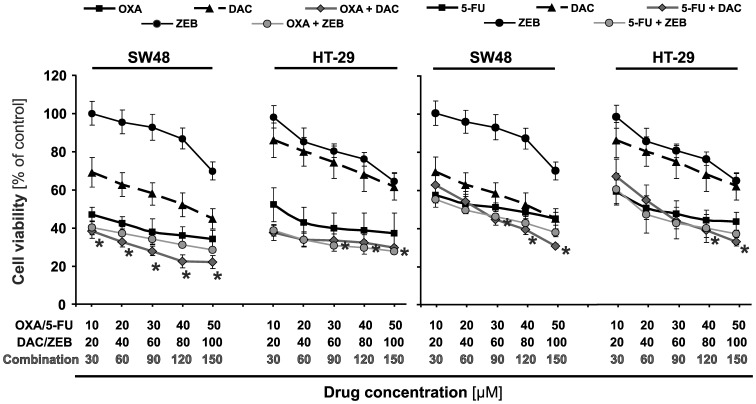
Effects of chemotherapeutic agents and DNMT inhibitors on cell viability of colorectal cancer cell lines. The cells were treated singly or in combinations with the indicted doses of the agents for 72; 5-FU, 5-fluorouracil; DAC, decitabine; and ZEB, zebularine. Each point represents the mean ±SD (n = 5), asterisks indicate a significance at *P*<0.05 for comparison with oxaliplatin or 5-FU alone.

As the first step, we examined the effect of the agents applied alone on SW48 and HT-29 cell viability. The cells were treated with 10 to 100 μM of the evaluated agents. The results of MTT cell viability assay indicated that CRC cells incubated with oxaliplatin and 5-FU showed the most potent inhibition of cell growth following 72 h incubation time (Figure 1). The inhibitory effects of chemotherapeutics were dose-dependent and the correlation coefficients (estimated from the inhibitory dose-response curves) were above 0.9. Demethylating agents, decitabine and zebularine, have demonstrated different modes of inhibition: decitabine was already inhibitory at starting concentration (20 μM) and zebularine showed inhibitory effect from 80 and 40 μM for SW48 and HT-29 cells, respectively.

We tested also the treatment of chemotherapeutic agents in combination with DNMTi on CRC cells survival (Figure 1). Decitabine potentiated the inhibitory effects of oxaliplatin at the concentration from 20 μM up to 100 μM, whereas its impact on 5-FU activity starts at 80 μM. Zebularine tested at concentrations up to 100 μM slightly potentiated the inhibitory effects of either chemotherapeutics (Figure 1).

**Figure 2:**
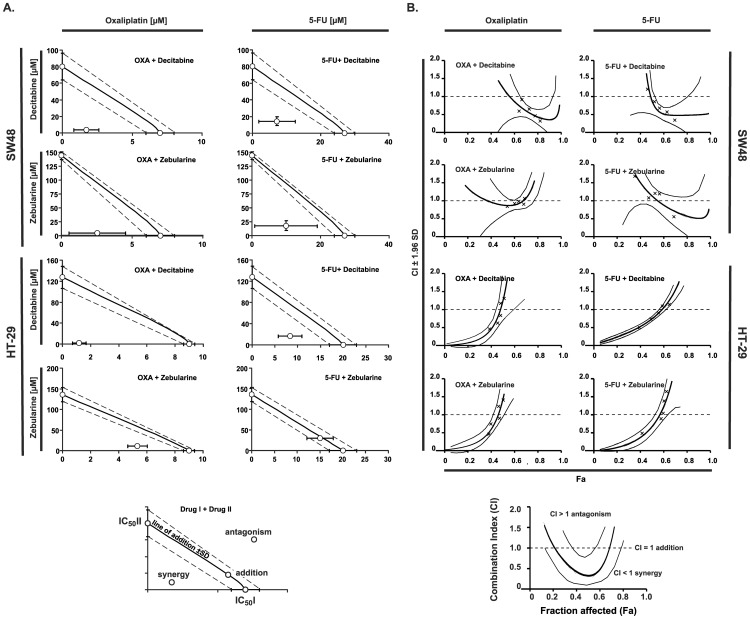
Interactions of standard chemotherapeutics with DNMT inhibitors in the human colorectal cancer cell lines SW48 and HT-29. **A.** Isobolograms at a 50% effect level. Concentrations of particular drugs are indicated on x and y axis. The isobolograms were constructed by connecting the IC_50_ values of demethylating agents (on the ordinate) with the IC_50_ of oxaliplatin or 5-FU plotted on the abscissa. When the doses of two agents in combination are lower or higher than the additive doses, the synergy or antagonism is present, respectively. **B.** Combination index values (CI) with a 95% confidence interval at all effect levels as calculated by the CalcuSyn software. A CI value significantly less than 1 indicates synergy, a CI value insignificantly different from 1 indicates addition, and a CI significantly higher than 1 indicates antagonism.

The type of drug interactions of two evaluated compound groups were analyzed with the help of isobolographic and median effect methods. The isobolograms show results for single 50% effect level and indicate that interaction of decitabine with chemotherapeutic agents results in synergistic effect, whereas administration of zebularine with oxaliplatin or 5-FU results in synergistic or slightly additive effects (Figure 2A).

Analysis performed by means of median effect method confirmed that the combination of decitabine and chemotherapeutic agents in both cell lines produced synergistic effects at 50% cell kill level (Fa  =  0.5), achieving CI (Combination Index) values < 0.9 (Figure 2B).

Combination of zebularine and chemotherapeutics for Fa  =  0.5 indicated slight synergistic or additive effects.

### Analysis of DNA damage

To elucidate the mechanism of apoptosis, we analyzed the phosphorylation status of proteins being of major signaling checkpoints in response to DNA damage.

Upon sensing the DNA damage, series of cellular signaling events designed to maintain the integrity and proper function of cells and biological pathways are involved. The proteins chosen for testing by Western blotting initiate cascades of events that block cell cycle progression either to allow time to repair damaged DNA or activate cell death pathways if too much damage has been incurred. Phosphorylated histone H2A.X localizes to the sites of the DNA damage and activates the ATM/ATR kinases, the central mediators of the DNA damage response. In turn, ATM/ATR kinases initiate cascades, which inhibit progression into mitosis through the activation of the Chk kinases (Chk1 for ATR and Chk2 for ATM) or tumor suppressor protein p53, leading to either cell cycle arrest and DNA repair or apoptosis through regulation of p53 downstream effectors. BRCA1, as the guardian of genomic stability, is also phosphorylated by ATM, ATR, and Chk2 in response to DNA damage and can co-localize with other proteins at DNA damage sites [15].

**Figure 3:**
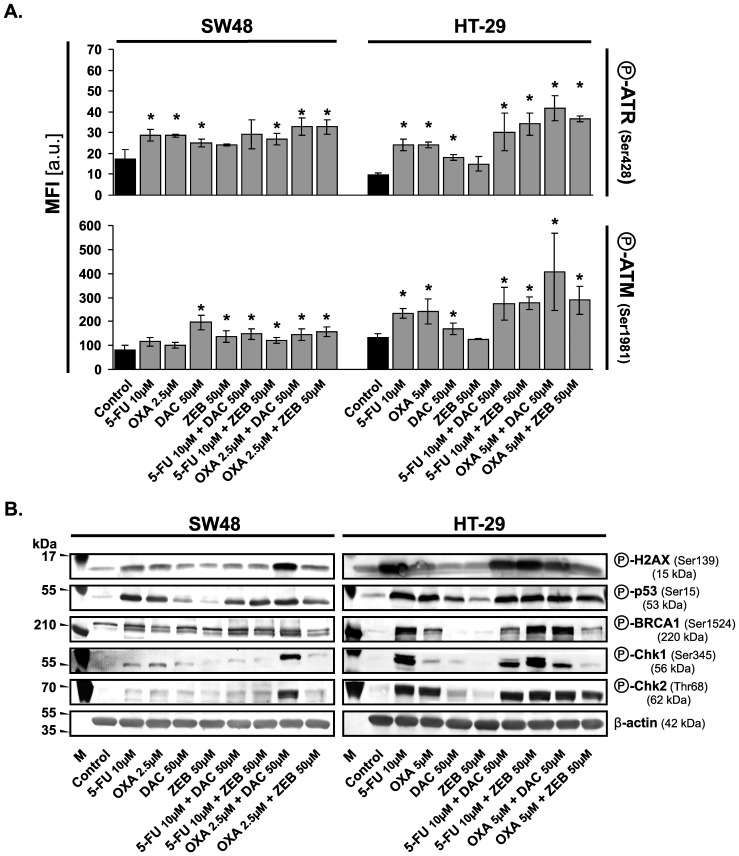
Combinations of chemotherapeutics with DNMTi agents induce major signaling checkpoints in response to DNA damage. **A.** Quantification of ATR and ATM phosphorylation level following 72(MFI) was used. Data represented the mean ±SD (n = 3). Significant difference at *P*≤0.05 is indicated by an asterisk (*). **B.** Analysis of protein phosphorylation levels using Western blotting method. The detection of β-actin was used as a gel loading control. OXA, oxaliplatin; 5-FU, 5-fluorouracil; DAC, decitabine; ZEB, zebularine; a.u., arbitrary units.

We observed a significant phosphorylation of histone H2A.X at Ser139 in response to chemotherapeutic agents and their combinations with DNMTi, especially in HT-29 cells. Lower levels of γH2A.X (phospho-H2A.X) were observed in cells treated with DNMTi agents only. We also observed elevated levels of phosphorylation of proteins as ATR at Ser428, ATM at Ser1981, p53 at Ser15, BRCA1 at Ser1524 as well as kinases Chk1 and Chk2 at Ser345 and Thr68, respectively (Figure 3A and B). Phosphorylation status of kinases Chk1 and Chk2 was more pronounced in HT-29 then in SW48 cells. In SW48 cells, phosphorylation levels of Chk1 and Chk2 kinases was higher after the combined treatment with oxaliplain and decitabine as compared to the treatment with both chemotherapeutics and DNMTi applied singly (Figure 3B).

### Combinations of chemotherapeutics with DNMTi influence the cell cycle progression and induce apoptosis

In the control culture, the percentage of cells in each phase of the cell cycle was stable, whereas the tested compounds had various effects on cell cycle progression and apoptosis. Treatment with the chemotherapeutics resulted in a consistent increase in the number of cells in the S phase at the expense of G1 phase. Decitabine arrested the cell cycle in the G2/M or S phase in the SW48 and HT-29 cells, respectively. Zebularine did not exert statistically significant changes in both cell lines, as compared to the controls.

**Figure 4:**
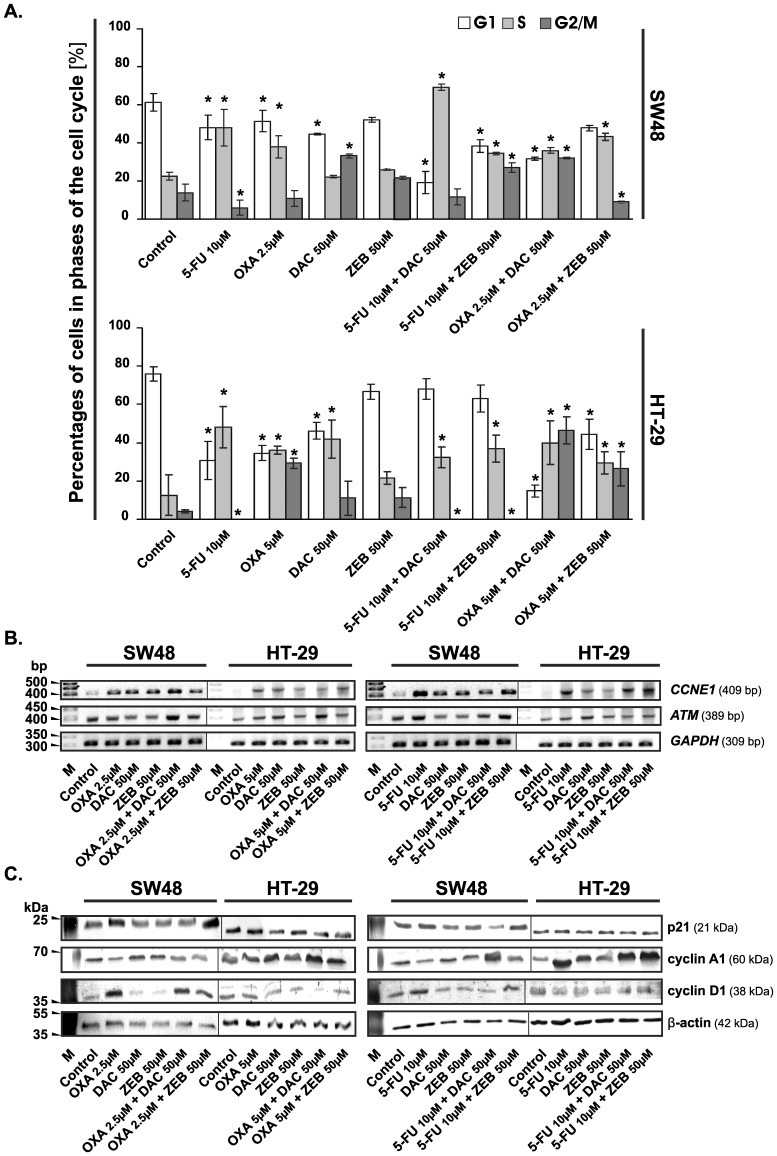
Chemotherapeutic agents, DNMTi and their combinations influence the cell cycle progression of colorectal cancer cells. **A.** Changes in the cell cycle distribution of SW48 and HT-29 cells after 72 h of treatment with the evaluated agents. The cells were stained with propidium iodide (PI) and then analyzed by flow cytometry. The percentage of cells in each phase of the cell cycle was determined using ModFit LT™ (version 3.0). Each bar represents the mean ±S.D. (n≥4). Significant difference at *P*<0.05 are indicated by asterisk (*). **B.** Analysis of *CCNE1* and *ATM* mRNA levels by semi-quantitative RT-PCR method after 72 h incubation of CRC cells with chemotherapeutic agents, DNMTi and their combinations at concentrations as indicated. M, marker [bp]; *GAPDH*, transcript encoding glyceraldehyde-3-phosphate dehydrogenase, a constitutively expressed gene, used as an internal control. **C.** Western blotting analysis of the cell cycle regulatory proteins. The β-actin was used as a gel loading control. OXA, oxaliplatin; 5-FU, 5-fluorouracil; DAC, decitabine; ZEB, zebularine.

The combination of decitabine with oxaliplatin or 5-FU induced a cell cycle arrest at the S/G2/M phase boundary and at the S phase, respectively, in both cell lines (Figure 4). The combination of zebularine with oxaliplatin increased percentage of the HT-29 cells at the S/G2/M phase boundary, whereas the combination of zebularine with 5-FU increased percentage of these cells in S phase from 12% to 36% as compared with control. In the case of the SW48 cells, the combinations of zebularine with chemotherapeutics caused the arrest of cells in the G2/M or S phase (Figure 4A).

**Figure 5:**
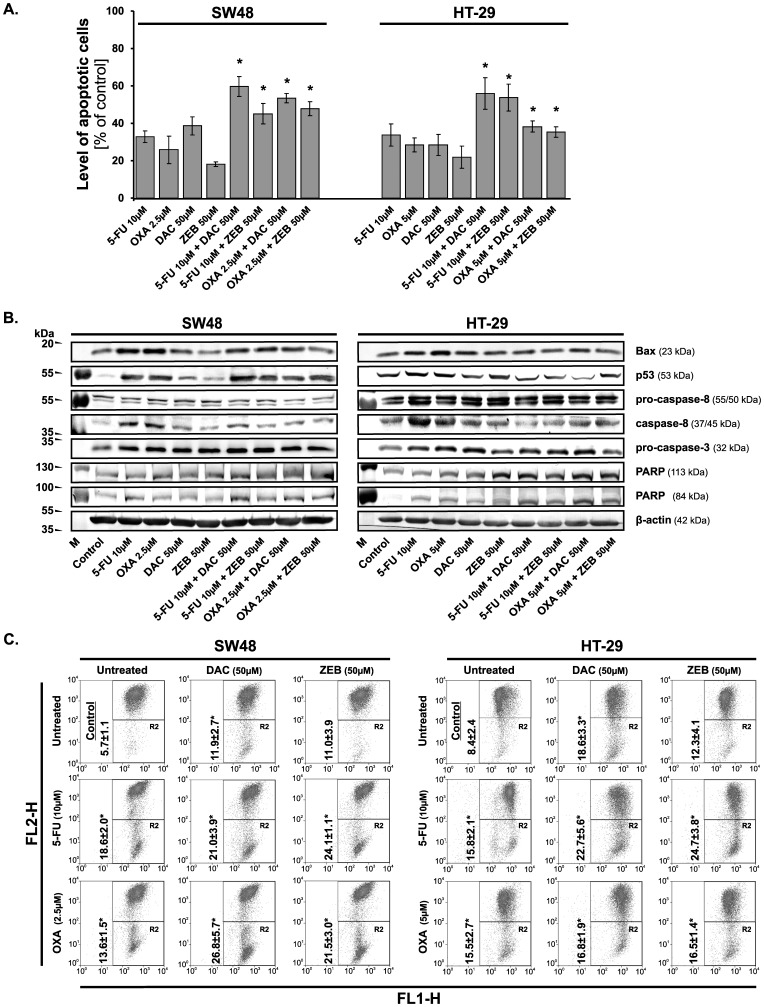
Induction of apoptosis in colorectal cancer cells following 72 h incubation with chemotherapeutic agents, DNMT inhibitors and their combinations. **A.** Detection of apoptosis by the annexin V-fluorescein isothiocyanate (FITC)/porpidium iodide (PI) analysis. Data are expressed as the means ±S.D. (n≥4). An asterisk (*) indicates that the induction of apoptosis by the evaluated agents was significant in comparison with the combination of chemotherapeutic agents and DNMT inhibitors *versus* chemotherapeutic agents applied alone (*P*<0.05). **B.** Western blotting analysis of pro-apoptotic protein levels. The β-actin was used as a gel loading control. **C.** Representative cytograms of flow cytometry experiments demonstrating changes in Δ*Ψ_m_* in CRC cells lines after 72 h of incubation with chemotherapeutic agents, DNMT inhibitors and their combinations. Cells were stained with JC-1. Cells in the R2 quadrant were counted as cells deprived of mitochondrial membrane potential. An asterisk (*) indicates a significant difference between experimental groups and control group at *P*<0.05. OXA, oxaliplatin; 5-FU, 5-fluorouracil; DAC, decitabine; ZEB, zebularine.

The analysis of gene expression revealed that the combination of decitabine with oxaliplatin, as compared to oxaliplatin alone, increased mRNA level of *ATM* in both cell lines as well as *CCNE1* in SW48 cell line (Figure 4B). The combinations of demethylating agents with 5-FU showed a similar tendency, but at a lower level than the combinations with oxaliplatin. The analysis of proteins specific for cell cycle progression revealed that the combination of DNMTi with chemotherapeutics, as compared with oxaliplatin/5-FU alone, increased the level of cyclin A1 and protein p53 and decreased the level of cyclin D1 (but not in the SW48 cells) (Figure 4C and 5B). The combination of zebularine with chemotherapeutics increased the level of p21 in SW48 cells (Figure 4C).

Since the prolonged treatment with chemotherapeutics or DNMTi might induce cell death, the early apoptotic marker was thus analyzed. For this purpose we used annexin V, which recognizes phosphatidyl serine (PS). During induction of apoptosis, membrane asymmetry is lost and translocation of PS from intracellular to external leaflet of the plasma membrane takes place. As we expected, the treatment of the CRC cells with oxaliplatin or 5-FU alone induced apoptosis in ∼30% of the cells, while combinations of the evaluated agents generally increased the number of apoptotic cells from ∼45% to 60% (Figure 5A) with the exception of the HT-29 cells incubated with demethylating agents and oxaliplatin together. In this case, the induction of apoptosis was at a lower level than for other combinations, but still statistically significant as compared with oxaliplatin applied alone. In addition, the induction of apoptosis in the CRC cells by combination of chemotherapeutics with demethylating agents was confirmed by changes at the protein level of pro-caspase-3 and -8. The proteolytic cleavage of the poly (ADP-ribose) polymerase (PARP) was augmented following incubation of cells with chemotherapeutics and demethylating agents (Figure 5B).

### The changes in mitochondrial membrane potential (ΔΨ_m_)

During early apoptosis the disruption of mitochondrial membrane potential Δ*Ψ_m_* may occur resulting in rapid collapse of the electrochemical gradient. In this work, we explored the effect of DNMTi, chemotherapeutics or their combinations on Δ*Ψ_m_* by staining the cells with JC-1 dye. The analysis of cytograms showed that the control cells emitted the red fluorescence due to high Δ*Ψ_m_*, whereas the cells treated with chemotherapeutics and DNMTi agents exhibited increased membrane depolarization, detected by green fluorescence, and the effect was sustained (oxaliplatin + DNMTi in HT-29 cell line) or stronger when cells were co-incubated with these compounds (Figure 5C).

## Discussion

In the 90s it has been confirmed that the CRC results not only from accumulation of genetic mutations but also as a consequence of epigenetic alterations of the cellular genome that transforms a normal glandular epithelium into adenocarcinoma. It is well established that the most extensively characterized epigenetic alteration in CRCs is gene promoter hypermethylation, which occurs in CpG islands that are often present at the 5' region of approximately 60% of the genes. This phenomenon results from the increased activity of the DNMT enzyme whose overexpression is a hallmark of almost all the transformed cells [16]. A growing number of genes that are expressed in the colon have now been shown to be hypermethylated and silenced in colorectal cancer. These include genes involved in cell-cycle control, growth, differentiation, angiogenesis, adhesion, metastasis or DNA repair [17]. Since epigenetic modifications are potentially reversible, the idea was arisen that the employment of DNMT inhibitors may improve the treatment of CRCs, especially since the classic chemotherapeutic agents are of limited efficiency in the colorectal cancer treatment. Therefore developing of some new strategies for the treatment of such tumors is a challenge to oncology [18]. That is why we have presented the results of *in vitro* study concerning interactions of standard cytotoxic drugs, 5-FU and oxaliplatin [19], with DNA methyltransferase inhibitors (DNMTi) such as decitabine and zebularine.

DNMTis have recently gained a lot of attention as agents inhibiting cancer growth including colorectal carcinoma [20]. However, the role of their therapeutic potential may be properly evaluated only in combination with classic agents used in the CRC treatment, because all new therapeutic agents are introduced as add-on options. Since 5-fluorouracil and oxaliplatin constitute the backbone treatment of CRC [19], we investigated the effects on CRC cells survival adding decitabine or zebularine - the DNA methyltransferase inhibitors - to these chemotherapeutics.

We have confirmed under *in vitro* conditions that the studied agents given alone are able to induce the growth inhibition of the cancer cells with comparable order of potency calculated on the basis of the percentage of cell survival (Figure 1). Moreover, we have found an increased inhibition of CRC cells growth after treatment with the anticancer agents applied in combination (Figure 1). The isobolograms, constructed on the basis of IC_50_ values, indicated synergistic or additive interactions between chemotherapeutics and DNMTi agents. Decitabine had the most favorable interactions (synergy) in combination with chemotherapeutics in both cell lines; whereas interaction analysis for zebularine showed moderate synergistic or additive effects and the best results were achieved for combination with oxaliplatin (Figure 2).

The interactions observed between both DNMTi agents and chemotherapeutics were dose- and cell line-dependent. The synergy between evaluated chemotherapeutics and decitabine was seen in the SW48 and HT-29 cell lines as well as in the Colo-205 cells, as previously reported [21]. The sensitivity of CRC cells in combination of zebularine with chemotherapeutics was different and dependent on cell line. For this combination, synergism and addition as well as previously presented antagonism were observed [21]. Moreover, reactions of the CRC cells to zebularine and its combinations were a little weaker. It appears that the two DNA methyltransferase inhibitors belonging to the same class of agents influence the inhibitory effects of chemotherapeutics on CRC cells growth in a qualitatively different manner. These differences may be related to their structure that exerts a direct effect on the survival of CRC cells [4]. The same observation was made in leukemic cells by Flotho *et al.* [22], who have shown that DNMTis; such as azacitidine, decitabine and zebularine; produce distinct patterns of gene induction and repression and that this diversity is related to the differences in the structure and cellular pharmacology among DNMT-inhibiting cytosine nucleoside analogues. Decitabine has nitrogen in the place of carbon at position 5 of the pyrimidine ring, but zebularine does not. In spite of that, both agents are incorporated into DNA but before incorporation the substances require phosphorylation to render the respective nucleotide forms. Decitabine is converted by deoxycytidine kinase, whereas zebularine is a substrate for uridine-cytidine kinase [22], [23], [24]. The inhibitory activity of zebularine is not specific for DNMTs. It is also a strong inhibitor of cytidine deaminase [25], [26], therefore most of the drug may be sequestered by the enzyme, lowering thereby the effective concentration of the drug. Another explanation is that the inhibition of cytidine deaminase by zebularine increases cellular concentration of cytidine and deoxycytidine resulting in competitive inhibition of zebularine [27]. In addition, the kinetics of activation/inactivation and intracellular half-life of metabolites differ among different cytosine analogues.

Generally, decitabine and zebularine possess ability to bind covalently with DNMT thus obstructing DNA synthesis and, in this way, leading to the cell death. Additionally, decitabine may also induce DNA damage through structural instability at the site of incorporation [28], [29]. Our results reviled that the potential therapeutic effect of DNMTi agents, especially decitabine, may be enhanced by 5-FU or oxaliplatin. The former, as an antimetabolite, can impair replication fork progression by becoming incorporated into the DNA, whereas the latter induces DNA lesions by interstrand cross-links resulting in replication stress. Therefore, we decided to evaluate whether DNA damage process took place in the cells after treatment with chemotherapeutics, DNMTi and their combinations. It was confirmed by the phosphorylation analysis of histone H2A.X, a member of a histone H2A family. H2A.X becomes phosphorylated at Ser139 by ATM/ATR kinases to form γH2A.X, the earliest indicator of DNA damages [30], [31]. In the current study, we found that the level of γH2A.X was strongly increased, especially in HT-29 cells. Our results show that chemotherapeutics as well as their combinations with DNMTi may activate ATM/ATR kinases pathways (Figure 3) leading, in consequence, to perturbations in the cell cycle progression and/or apoptosis induction. This finding was confirmed by further studies discussed below.

Combinations of DNMTi with chemotherapeutics led to CRC cell cycle arrest in the S/G_2_/M transition or S phase, when compared to the control cycle and the reduction of cell number in the G1 phase (Figure 4). This was accompanied by increased mRNA level of *ATM*, as well as phosphorylation level of ATM at Ser1981. Furthermore, we observed the elevated level of ATR phosphorylated at Ser428. Activation of both kinases suggests that DNA double-stranded breaks (DBS) as well as single-stranded DNA (ssDNA) are formed [32]. The ssDNA is present at processed DSB ends but also at stalled replication forks. Phosphorylation of ATM and ATR kinases induces activation of Chk2 and Chk1 kinases, respectively, which in turn induces *via* phosphorylation the degradation of Cdc25A and C and phosphorylation of p53 protein [33]. We observed elevated levels of Chk2 and Chk1 phosphorylated at Thr68 and Ser345, respectively (Figure 3). This might indirectly confirm down regulation of Cdc25A and C, the protein tyrosine phosphtases, which are downstream targets of both kinases. Additionally, we observed a higher level of cyclin A probably connected with down regulation of Cdc25A and elevated phosphorylation of BRCA1 at Ser1524, which is essential for activating the Chk1 kinase and finally G_2_/M arrest (Figure 3) [34].

It is well known, that once the cells accumulate the excess of DNA damages that overwhelm their capacity of repairing the mechanisms for selective elimination of such cells are activated. Indeed, we have observed that simultaneous administration of the tested agents increased the apoptotic response of the cells (Figure 5A). The interaction among the agent combinations led to augmentation or maintaining of the protein levels of caspase-8 and p53 as well as the level of pro-caspase-3 dependently of the cell line studied (Figure 5B). Thus, the cytotoxic effect of DNMT inhibitors and chemotherapeutics may result from apoptosis induced by internal and slightly external signals. In fact, the possible involvement of mitochondrial pathway was confirmed by the finding that the agent combinations (with the exception of oxaliplatin + zebularine in HT-29 cells) strongly induced the disruption of Δ*Ψ_m_* (Figure 5C). Furthermore, the activity of these combinations was manifested by the cleavage of PARP protein into 84 kDa and 25 kDa fragments, which facilitates cellular disassembly and also serves as a marker of cells undergoing apoptosis (Figure 5B).

The results obtained in the analyzed cells lines are in some cases different, however the main observations and final conclusions are similar. In our opinion, the differences in the response of both cell lines may depend on genetic and epigenetic background of the cells. These cell lines carry a BRAF mutation (Colon Cancer Panel 2, BRAF; ATCC No. TCP-1007), but the HT-29 cells also carry a mutated *p53* gene (G→A mutation at codon 273 resulting in an Arg→His substitution, R273H) causing overproduction of a pathogenic form of p53, while SW48 cells have a wild-type allele. Since this additional mutation may cause higher sensitivity to such drug combinations, it may also be responsible for observed, in some cases, more pronounced response of HT-29 cells.

On the other hand, the SW48 cell line is known to harbor more of the epigenetic changes such as hypermethylation of CpG islands in the promoter regions leading to inappropriate silencing of some genes [34]. Moreover, both cell lines differ in global DNA methylation levels. Mossman *et al*. [35] have shown that HT-29 cell line displayed a lower level of global DNA methylation than SW48 cell line and that following the treatment of cells with decitabine, a substantial decrease of genomic DNA methylation was observed. The decrease in global methylation in SW48 cell line was greater than 50%, whilst the decrease in HT29 cell line was not so extensive. The authors also reviled that reduction of methylation at specific gene CpG islands was significantly less effective [35]. For this reason, we have also analyzed the methylation status of selected genes such as *p16^INK4a^*, *APC*, *p14^ARF^*, *DAPK* and *MLH1* using methylation-specific PCR (MSP) (data not shown) and the obtained results were comparable to those published by Deng *et al.* and Lind *et al.* [36], [37]. Among both cell lines tested, the biallelic methylation of promoter region was observed in *p16^INK4a^* and *MLH1* genes, whereas the biallelic methylation of *p14^ARF^* gene was observed only in SW48 cell line. Unfortunately, our studies did not confirm that the application of DNMTi restores the expression of the evaluated genes and that it is the way by which both agents might enhance the action of chemotherapeutics. Perhaps such results are linked to the dosage levels of the compounds tested as well as the incubation time. Most results presented by other authors, who observed the reactivation of silenced genes after decitabine or zebularine application, were conducted for longer time duration, i.e. up to 5–8 days, and doses of the compounds were much lower [8], [38]. Nevertheless, our results clearly show that DNMTi application can bring benefits also in methylation-independent manner and that their action relies on cytotoxic properties in the applied range of doses.

The mechanism of positive interactions between DNMT inhibitors and chemotherapeutic agents is related to triggering the cascade of events leading finally to apoptosis. Such augmentation suggests that the cytotoxic effects of classical chemotherapeutics used in the treatment of colorectal carcinoma could be obtained at lower doses and with higher probability for inducing cancer cell death when combined with DNMT inhibitors. It is also known that the abnormal methylation of promoter regions of regulatory genes is commonly associated with cancer development. Therefore, the methylation inhibitors such as decitabine or zebularine may reactivate the silenced tumor suppressor genes [39] responsible for controlling the cell cycle progression and induction of apoptosis, and in this way improve the effectiveness of anticancer therapy with chemotherapeutic agents.

In summary, we have demonstrated that DNMT inhibitors, in spite of the less potent action of zebularine, may exert a positive interaction with oxaliplatin and 5-FU, the standard chemotherapeutics used in cancer treatment, potentiating the inhibition of CRC cell survival *in vitro*. It seems very likely that in the case of zebularine a more favorable response could be achieved with higher doses than those presented here, since the toxicity of this agent was shown to be minimal [27]. Even so, the obtained results provide a rationale to continue research studies that could help develop more effective treatments using methylation inhibitors together with standard chemotherapeutics as a new hope in the colorectal cancer therapy.
