# Correction: DNA Methyltransferase Inhibitors Improve the Effect of Chemotherapeutic Agents in SW48 and HT-29 Colorectal Cancer Cells

**DOI:** 10.1371/journal.pone.0106142

**Published:** 2014-08-18

**Authors:** The PLOS ONE Staff

The authors are issuing this Correction to address concerns about Figure 4B of this article.

**Figure 4:**
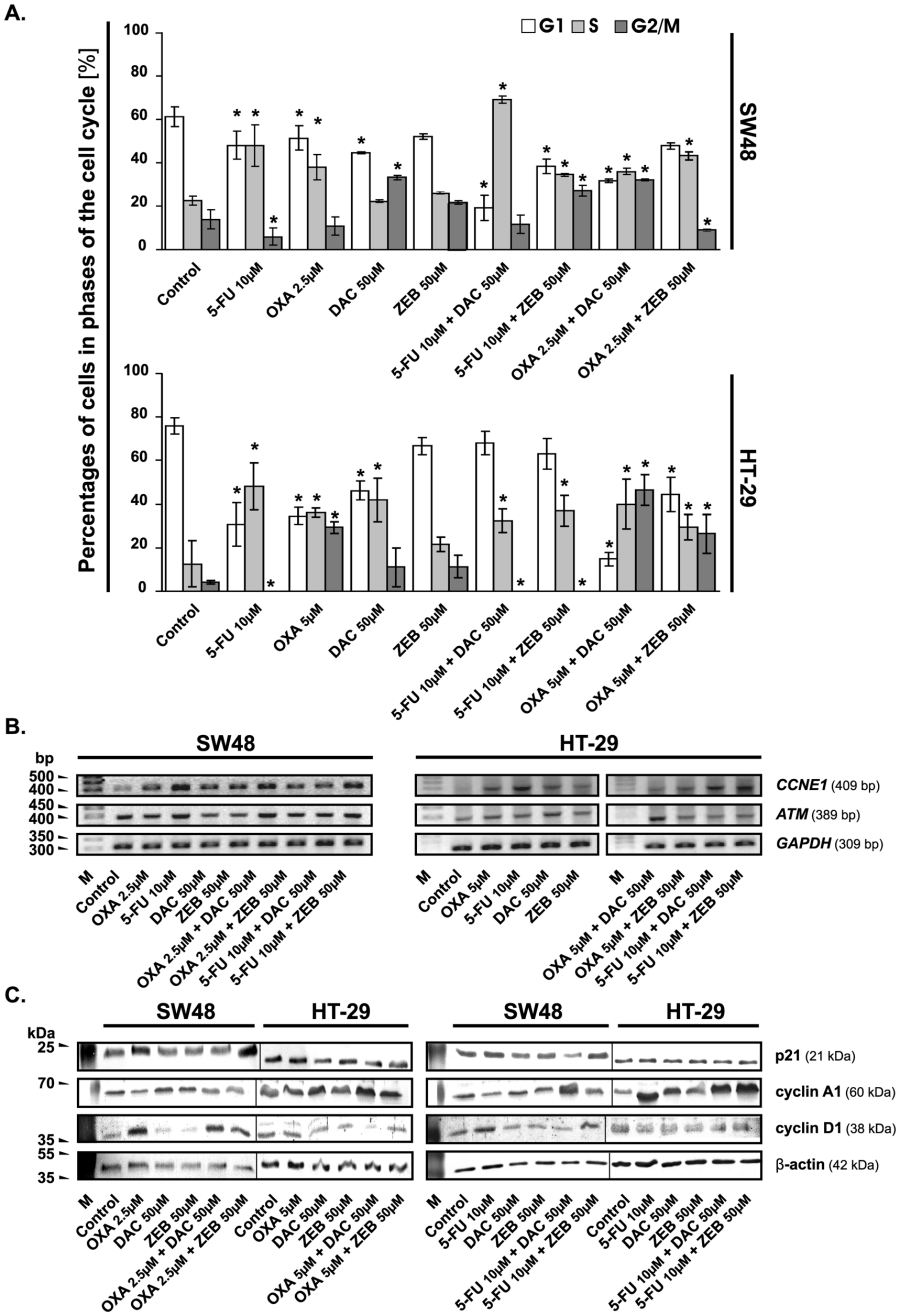
Chemotherapeutic agents, DNMTi and their combinations influence the cell cycle progression of colorectal cancer cells. **A.** Changes in the cell cycle distribution of SW48 and HT-29 cells after 72 h of treatment with the evaluated agents. The cells were stained with propidium iodide (PI) and then analyzed by flow cytometry. The percentage of cells in each phase of the cell cycle was determined using ModFit LT™ (version 3.0). Each bar represents the mean ±S.D. (n≥4). Significant difference at P<0.05 are indicated by asterisk (*). **B**. Analysis of CCNE1 and ATM mRNA levels by semi-quantitative RT-PCR method after 72 h incubation of CRC cells with chemotherapeutic agents, DNMTi and their combinations at concentrations as indicated. M, marker [bp]; GAPDH, transcript encoding glyceraldehyde-3-phosphate dehydrogenase, a constitutively expressed gene, used as an internal control. **C**. Western blotting analysis of the cell cycle regulatory proteins. The β-actin was used as a gel loading control. OXA, oxaliplatin; 5-FU, 5-fluorouracil; DAC, decitabine; ZEB, zebularine.

Lanes 1, 2, 4, and 5 of SW48 as well as HT-29 represent molecular weight marker and mRNA levels of particular genes in mock-, DAC- and ZEB-treated cells, respectively. The experimental conditions for each cell line presented in Figure 4B were tested in the same experiment, therefore the lanes are the controls for both OXA- (left panel) and 5-FU-containing (right panel) conditions. Since the authors wanted to keep the same order of the samples as in Figure 4C to easily compare the effects of OXA and 5-FU and their combinations with DNMTi, the lanes 1, 2, 4, and 5 of both cell lines have been reused in the right panel; which was further a source of some concerns.

Additionally, it was found that panel for the loading control (GAPDH) in Figure 4B was inadvertently duplicated, probably during figure preparation.

The authors apologize for the inaccuracies in the presentation of Figure 4B. They have prepared a new Figure 4B preserving the original order of the samples and avoiding duplications and errors. The authors have also provided the original uncropped pictures of ethidium bromide/agarose gels scanned from their laboratory notebooks.

The results and conclusions are not affected by this correction.

## Supporting Information

File S1 Raw Blots for Figure 4b SW48.
(PDF)

File S2 Raw Blots for Figure 4b HT29.
(PDF)

## References

[1] Flis S, Gnyszka A, Flis K (2014) DNA Methyltransferase Inhibitors Improve the Effect of Chemotherapeutic Agents in SW48 and HT-29 Colorectal Cancer Cells. PLoS ONE 9(3): e92305 doi:10.1371/journal.pone.0092305 24676085 10.1371/journal.pone.0092305 PMC3967992

