# Supplementary figures and images for: Correction: DNA Methyltransferase Inhibitors Improve the Effect of Chemotherapeutic Agents in SW48 and HT-29 Colorectal Cancer Cells
Source: PLoS One. 2014 Aug 18;9(8):e106142. doi: 10.1371/journal.pone.0106142 (PMC4136874; doi:10.1371/journal.pone.0106142)

# SW48 cells

## CCNE1

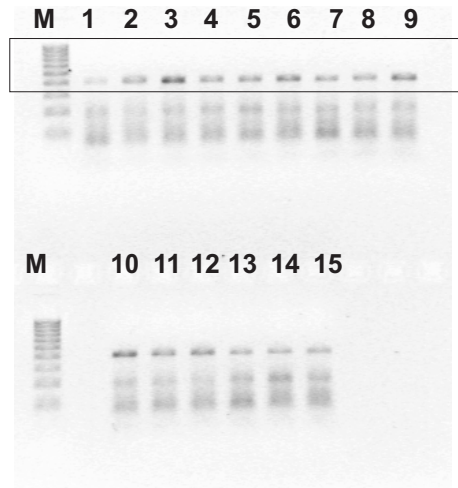

## ATM

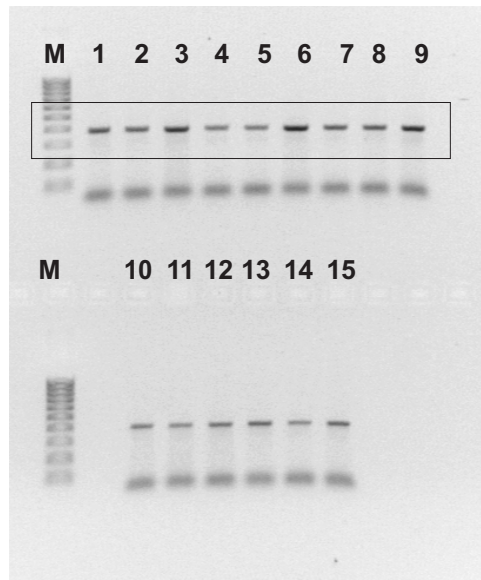

## GAPDH

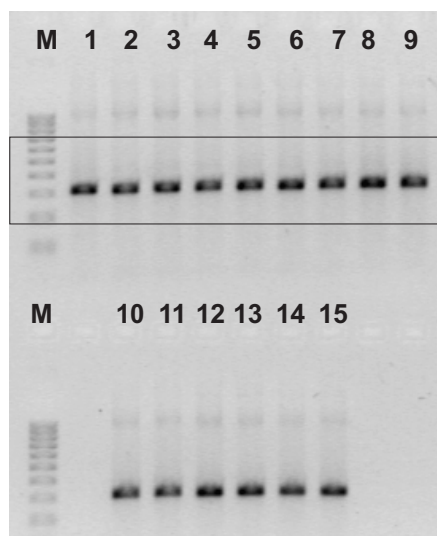

1. Control (not treated)
2. OXA
3. 5-FU
4. DAC
5. ZEB
6. OXA+DAC
7. OXA+ZEB
8. 5-FU+DAC
9. 5-FU+ZEB

Supplement: File S1 — Raw Blots for Figure 4b SW48. (PDF) [file pone.0106142.s001.pdf]
