# Supplementary material for: Correction: DNA Methyltransferase Inhibitors Improve the Effect of Chemotherapeutic Agents in SW48 and HT-29 Colorectal Cancer Cells
Source: PLoS One. 2014 Aug 18;9(8):e106142. doi: 10.1371/journal.pone.0106142 (PMC4136874; doi:10.1371/journal.pone.0106142)
Supplement: File S2 — Raw Blots for Figure 4b HT29. (PDF) [file pone.0106142.s002.pdf]

HT29 cells

CCNE1 HADC1

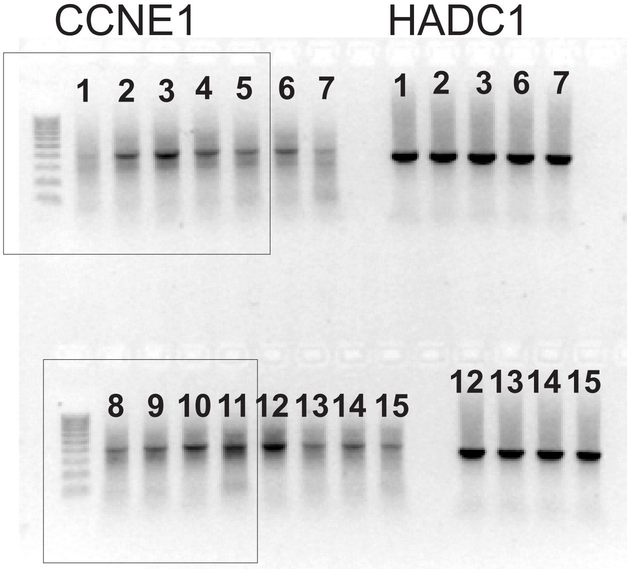

ATM

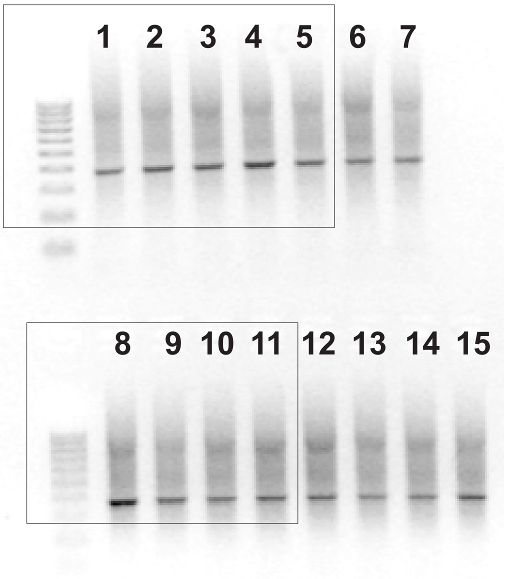

GAPDH

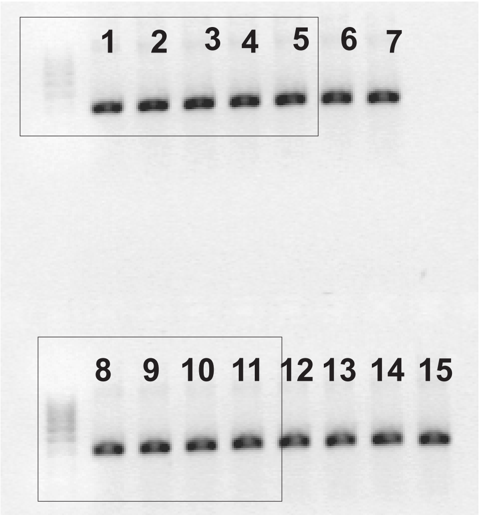

1.Control (not treated)
2. OXA
3. 5-FU
4. DAC
5. ZEB
8. OXA+DAC
9. OXA+ZEB
10. 5-FU+DAC
11. 5-FU+ZEB
